# Canonical Source Reconstruction for MEG

**DOI:** 10.1155/2007/67613

**Published:** 2007-08-06

**Authors:** Jérémie Mattout, Richard N. Henson, Karl J. Friston

**Affiliations:** ^1^INSERM U821, Dynamique Cérébrale et Cognition, Lyon, France; ^2^MRC Cognition and Brain Sciences Unit, Cambridge CB2 7EF, UK; ^3^The Wellcome Trust Centre for Neuroimaging, University College, London WC1N 3BG, UK

## Abstract

We describe a simple and efficient solution to the problem of reconstructing electromagnetic sources into a canonical or standard anatomical space. Its simplicity rests upon incorporating subject-specific anatomy into the forward model in a way that eschews the need for cortical surface extraction. The forward model starts with a canonical cortical mesh, defined in a standard stereotactic space. The mesh is warped, in a nonlinear fashion, to match the subject's anatomy. This warping is the inverse of the transformation derived from spatial normalization of the subject's structural MRI image, using fully automated procedures that have been established for other imaging modalities. Electromagnetic lead fields are computed using the warped mesh, in conjunction with a spherical head model (which does not rely on individual anatomy). The ensuing forward model is inverted using an empirical Bayesian scheme that we have described previously in several publications. Critically, because anatomical information enters the forward model, there is no need to spatially normalize the reconstructed source activity. In other words, each source, comprising the mesh, has a predetermined and unique anatomical attribution within standard stereotactic space. This enables the pooling of data from multiple subjects and the reporting of results in stereotactic coordinates. Furthermore, it allows the graceful fusion of fMRI and MEG data within the same anatomical framework.

## 1. INTRODUCTION

Source reconstruction in neuroimaging, particularly PET and fMRI, is usually into a standard anatomical space (e.g., that defined by the Atlas of [1]). Reconstruction into a canonical space facilitates the formal or informal meta-analysis of findings in imaging neuroscience and provides a useful framework within which to define structure-function relationships. In PET and fMRI the construction of spatially normalized images comprises two distinct steps. First, the raw data are reconstructed into images of source activity within the subject's own anatomical space. Second, these data are then spatially normalized into a standard space using a template matching approach (e.g., [2]). For EEG and MEG, however, source reconstruction and spatial or anatomical normalization cannot be separated because the reconstruction depends upon the spatial configuration of sources.

The central idea, upon which this work is based, is to include anatomical variability in a forward model that links MEG responses to canonical sources. Specifically, the anatomical differences between a particular subject and a canonical subject (who conforms to the standard space) enter the forward model. Note that these differences are expressed in both cortical anatomy and in the geometrical and physical properties of other tissues (e.g., skull and scalp), through which electromagnetic fields propagate to the sensors. However, we restrict ourselves here to the effect of inter-subject variability in cortical anatomy, given that for MEG, spherical conductor models, which need not incorporate subject specific information about the head, generally provide a sufficiently good approximation compared with more realistic head models such as those using boundary element methods (BEM) see [3, 4]. In contradistinction, the inverse solution is highly sensitive to the source location and orientation, when defined by the cortical anatomy [5]. The nice thing about the approach used here is that spatial normalization becomes an implicit part of the inverse solution. In this paper, we describe how this can be implemented using fully automated procedures that are already in routine use and are freely available as academic software (see Software note).

The basic idea is to formulate a forward or generative model of how a specific subject's MEG data were caused and then invert this model using standard Bayesian techniques. We start with a canonical subject whose anatomy conforms to a predefined space; the MNI-space based upon the Talairach and Tournoux system [1]. This is the same space as used by the SPM software and, more generally, by the neuroimaging community when reporting fMRI and PET results. A canonical mesh is defined within this space, coding the position and orientation of dipolar sources. Warping the mesh to match the subject's anatomy creates a subject specific model. After warping, subject specific forward fields (i.e., a gain matrix) are computed using standard electromagnetic forward modelling procedures. In this paper, we use a single-sphere head model, fit to the template scalp surface. The resulting forward model has two components an anatomical component that displaces and reorientates the dipoles into subject specific anatomy and an electromagnetic component that projects the source activity to measurement space (i.e., channels). Reconstruction of the canonical sources corresponds to the inversion of this forward model, given some data. The conditional estimates of source activity can then be treated within a canonical space. In other words, the source activity is associated with the original mesh (before warping).

There are several advantages of the approach described in this paper. The primary advantage is that it allows for anatomically informed source reconstruction into a standard space that facilitates inter-subject pooling and standardized reporting of results. The second main advantage is that it does not entail the accurate extraction of a subject specific cortical surface. This means that the spatial constraints can be based upon any anatomical information, irrespective of whether its quality would support cortical surface extraction or not. Another advantage is that the estimation can proceed even in the absence of a subject's MRI. In this instance, the reconstruction assumes that the subject's anatomy was, in fact, canonical. A final advantage, which will be pursued in a subsequent paper, is that conditional uncertainty about the subject's anatomy can be handled gracefully during Bayesian inversion. It is worth noting that the two key methodologies, namely, estimating the mapping from canonical to subject specific anatomical space and Bayesian inversion of MEG forward models, are fully established and in routine use. Furthermore, because they are fully automated and deterministic, there is no need for human intervention, which renders the procedure totally reproducible.

The aim of this paper is first to motivate and to describe the operational details of a fully automated canonical source reconstruction. Second, we demonstrate, quantitatively, the performance of this inverse-normalized canonical mesh approach in comparison with (i) reconstructions based upon the subject's native mesh and (ii) the canonical mesh without any spatial transformation. In a later paper, we will use canonical reconstructions in a hierarchical model of multi-subject responses measured with EEG and MEG. This paper is restricted to the analysis of single subjects.

This paper is organized as follows. In Section 1 we review the theoretical aspects of the procedure. This entails a brief review of our Bayesian approach to conventional forward models. We then consider spatial normalization. Finally, we see how these two components are integrated to enable canonical source reconstruction. The second section is an empirical demonstration of the utility of the approach. Because the estimation scheme is Bayesian, we can use Bayesian model comparison to evaluate different models. This comparison rests on the log evidence or likelihood of the data given a particular model (having integrated out any dependencies on the model's parameters or hyperparameters). Put simply, we can quantify the likelihood of any given data set given one model, relative to another. Here, we compare three sorts of models: first, a baseline model where the electromagnetic model was based upon a canonical mesh without spatial transformation. The second model, used to explain the same data, incorporated anatomically informed spatial transformations of the canonical mesh. We also evaluated a gold-standard model where the cortical mesh was obtained from a cortical surface extraction, using the subject's MRI data. We hoped to show that including the spatial transformation in the reconstructions would yield a greater log evidence than for the baseline model, and that this log evidence was not significantly less than for the gold-standard model based upon the subject's cortical surface.

## 2. THEORY

### 2.1. Bayesian source reconstruction

In a series of papers [6, 7] we have described a Bayesian approach to inverting forward models for EEG and MEG. These forward models start with a subject specific cortical dipole mesh or three-dimensional grid, referred to as the subject's source space. This, in conjunction with the position of the sensors, is used to compute a Gain matrix *L* in the usual way, under quasistatic Maxwellian assumptions. The inversion of the ensuing electromagnetic forward model uses a hierarchical linear observation model and conforms to parametric empirical Bayes (PEB) using restricted maximum likelihood (ReML). The Bayesian aspect accommodates the regularization required for ill-posed inverse problems. The empirical aspect allows us to identify the ReML estimators of hyperparameters *λ* controlling multiple noise and prior covariance components, *Q_i_* ^(1)^ and *Q_i_* ^(2)^, respectively. The key advantage of this approach is that it can accommodate multiple priors in a principled and efficient way. Its efficiency stems from the fact that the ReML scheme estimates covariance components in low-dimensional sensor space, as opposed to high-dimensional source space.

The objective function used by this scheme is equivalent to the ReML objective function, which, as shown in [8], is identical to the (negative) variational free energy

**Figure 1:**
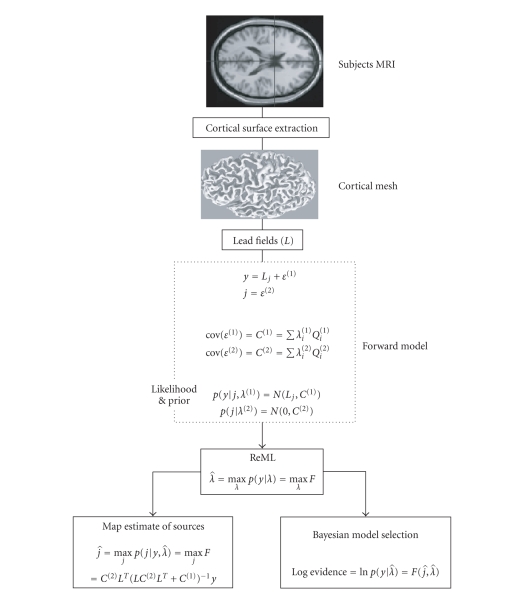
Bayesian inversion scheme.

(1) F=〈ln p(y∣j,λ)+p(j∣λ)−ln q〉q,

where *y* is the data, *j* are the source activities, and *q*(*j*) is their conditional or posterior density. Under Gaussian assumptions, when *F* is maximized; *q*(*j*) = *p*(*j* ∣ *y*,*λ*), and the (negative) free energy becomes the log likelihood of the model or its log evidence *F* → In*p*(*y* ∣ *λ*) [9]. We have shown how the log evidence can be used to compare and adjudicate among different models comprising different prior covariance components or different source configurations [7]. We use exactly the same approach below, to compare three different sorts of anatomical source models, each with slightly different configurations of a cortical mesh subtending the lead fields. Figure 1 provides a schematic that summarizes this Bayesian inversion scheme.

### 2.2. Spatial normalization

**Figure 2:**
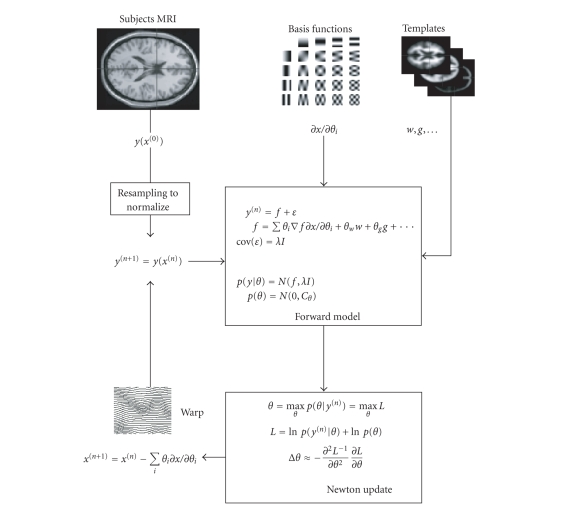
Spatial normalization scheme.

Spatial normalization is a term that refers to the warping or mapping of a subject specific image into a standard anatomical space. It is used routinely in fMRI and PET to enable inter-subject pooling. The parameters *θ*_i_ that define the transformation *x* ^(0)^ → *x* ^(*n*)^ are identified using a Bayesian scheme that incorporates constraints on the smoothness of the transformation [2]. *x*_i_^(*n*)^ represents the position of the *i* th control point after *n* iterations. In brief, the warping is parameterized in terms of spatial basis functions (in SPM, we use a discrete cosine set). These encode the change in position effected by each transform parameter *∂* *x*/*∂* *θ*_i_ The coefficients of these basis functions maximize their conditional probability (i.e., maximize the likelihood and prior density). The likelihood is computed using a forward model, which mixes several canonical templates and then warps them to predict the observed image. The mismatch between the warped mixture of templates and the observed image constitutes a prediction error. Under Gaussian assumptions this error gives the likelihood of the observed image, given the mixing and warping parameters. Rough transformations are penalized by appropriate shrinkage priors on the coefficients, formulated in terms of their covariance. The parameters are computed using a Newton method. The inverse of the template warping is applied to the image and the process iterated until convergence and the image is spatially normalized (see Figure 2 for a schematic).

Once the normalizing transformation has been identified, given some structural image it is usually applied to spatially normalize the subject's functional time series so that analysis can proceed in standard space. A full description of the assumptions and procedures entailed by spatial normalization can be found in a series of papers [10, 11]. Here, we do not use the spatial transformation to normalize reconstructed sources but to spatially *unnormalize* a canonical mesh to inform the forward model about how that subject's electromagnetic signals were generated. This simply involves applying the inverse spatial transformation *x* ^(*n*)^ → *x* ^(0)^ to the locations of the canonical mesh dipoles.

### 2.3. Canonical source reconstruction

**Figure 3:**
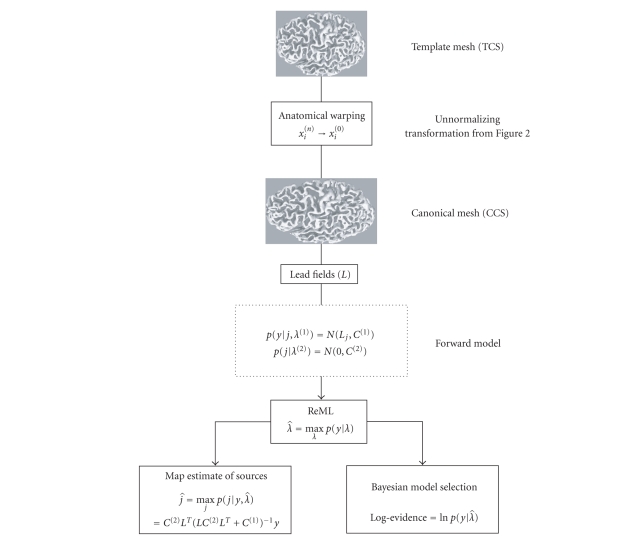
Overview of canonical source reconstruction.

Canonical source reconstruction is identical to our Bayesian source reconstruction (see Figure 1) with the addition of an anatomical component to the forward model. This component is the spatial transformation of a canonical cortical mesh to match the subject's anatomy using the inverse of the spatially normalising transformation (see Figure 3). After transformation, the subject specific mesh is used in the usual way to create an electromagnetic forward model that is inverted as described above. The evidence for this model that comprises both the anatomical and electromagnetic components can then be used to compare different models.

In the next section, we apply the above theory to both synthetic and real MEG data. Our primary goal is to ascertain the relative likelihoods of the different models considered. However, we also take the opportunity to demonstrate the procedure and provide a worked example of its application.

## 3. MODEL COMPARISON

### 3.1. Anatomical models

In what follows, we use the following acronyms for the meshes used by the models, which differ only in their anatomical information.

- SCS (subject's cortical surface) refers to the mesh obtained from cortical surface extraction, using the subject's structural MRI. This constitutes our gold standard in the sense it makes the least anatomical assumptions. The meshes were obtained using the BrainVISA ^1^ software [12, 13]. A “fine” mesh was used to generate the synthetic MEG data, while a “coarse” mesh was used to reconstruct the cortical activity, comprising 7204 and 4004 vertices, respectively.
- CCS (canonical cortical surface) refers to a subject specific canonical mesh obtained by applying an inverse spatial transformation to a template mesh in canonical space (the TCS). The transformation is derived by normalising the subject MRI as described in Section2.2.
- TCS (template cortical surface) refers to the (un-transformed) template mesh in canonical space. This model would be used typically when no structural MRI of the subject is available.

To build the TCS, a cortical mesh of a neurotypical male was extracted from his structural MRI, using BrainVISA. This furnished a high-density mesh, with a uniform discrete coverage of the grey/white matter interface. This mesh corresponds to the TCS currently available in the latest release of the SPM software package (see Software note). Here we use the TCS mesh downsampled to 4004 vertices, to match the SCS for source reconstruction. For any given mesh, each vertex location corresponds to a dipole position, whose orientation is fixed perpendicular to the surface. Note that our forward models, based on high-density meshes, could be replaced with low-density meshes with free dipole orientations to compensate for the loss of degrees of freedom implicit in reducing dipole number.

The single subject we considered here was a healthy female volunteer who participated in an MEG study of face perception. We chose a female to deliberately maximize the differences between subject and template anatomy.^2^ This enabled us to assess the effectiveness of the warping procedure, under a substantial anatomical distance between SCS and TCS. Furthermore, it induced a greater difference between the warped (CCS) and unwarped (TCS) cortical surfaces, whose influence on the ensuing reconstruction could be observed. Clearly, we anticipate formal and anecdotal replications of the analyses presented in this paper that will allow its conclusions to be generalized to the population of normal subjects.

The two anatomical models for this subject (SCS and CCS) as well as the template mesh (TCS) were compared in the context of simulations and real experiment. In all cases, the sensor locations were registered to source space and the gain matrix was computed using a single sphere-head model [14], fit to the template scalp mesh. The latter was obtained with BrainVISA and used to get the best fitting sphere to be used in the forward computation. As a consequence, the head model was common to each anatomical model and based on the template geometry. We are thus in the position to compare the models, based on their representation of the cortical anatomy only. Bayesian inversion of the ensuing forward model assumed independent channel noise and simple minimum norm priors (i.e., *Q* ^(1)^ and *Q* ^(2)^ were identity matrices). This corresponds to the classical minimum norm solution, although the relative weight of the likelihood and prior are optimized using ReML as opposed to the conventional *L* -curve heuristic. ReML has been shown to provide optimal hyperparameter estimates [6, 7], when compared to alternative schemes. Furthermore, this Bayesian inversion enables us to use log-normal hyperpriors on the hyperparameters and enforce a positive contribution of each variance component [9].

Although the log evidence reflects both goodness of fit and model complexity [15], the complexity term for each model was exactly the same. This is because the only difference between the models was in the location of the dipoles encoded by the cortical meshes. In short, the three models compared here match perfectly in terms of complexity and number of free parameters (degrees of freedom).

### 3.2. Analyses of real data

**Figure 4:**
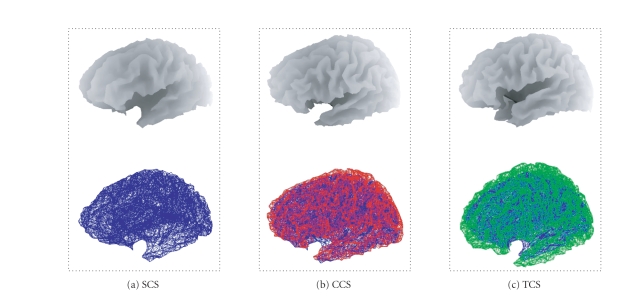
Surface rendering (upper row) and meshes (lower row) encoding the three cortical models: SCS (a), CCS (b), and TCS (c). CCS (red) and TCS (green) meshes are superimposed on the SCS mesh (blue).

The MEG dataset came from the female subject, who participated in a multimodal study on face perception (for description of paradigm see [16]). The subject made symmetry judgments on faces and scrambled faces. The MEG data were acquired on a 151-channel CTF Omega system at the Wellcome Trust Laboratory for MEG Studies, Aston University, England. The epochs (80 face trials, collapsing across familiar and unfamiliar faces, and 84 scrambled trials) were baseline corrected from − 100 milliseconds to 0 millisecond, averaged over trials and bandpass filtered (between 1 and 30 Hz). The subject's T1-weighted MRI was obtained at a resolution of 1× 1 × 1 mm^3^ . The subject's head shape was digitized with a 3D Polhemus Isotrak and was used to coregister the MEG sensor locations to anatomical space using a rigid-body (six-parameter) affine transformation. Figure 4 shows the three meshes SCS, CCS, and TCS defining the three models.

### 3.3. Results for real data

**Figure 5:**
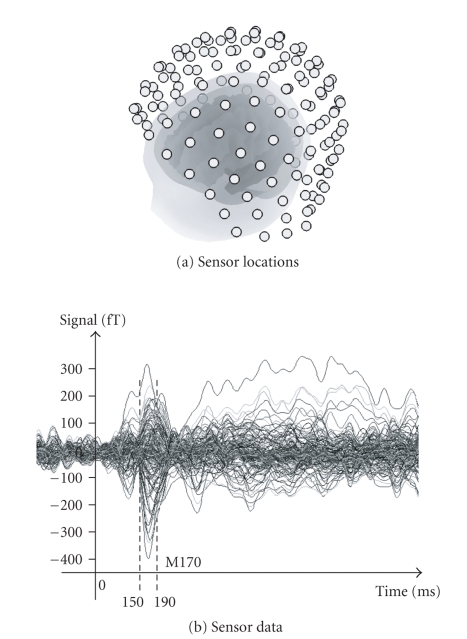
(a) Sensor locations coregistered with the subject's MRI-derived meshes of the cortical, skull, and scalp surfaces; (b) sensor data for the difference between faces and scrambled event related fields.

**Figure 6:**
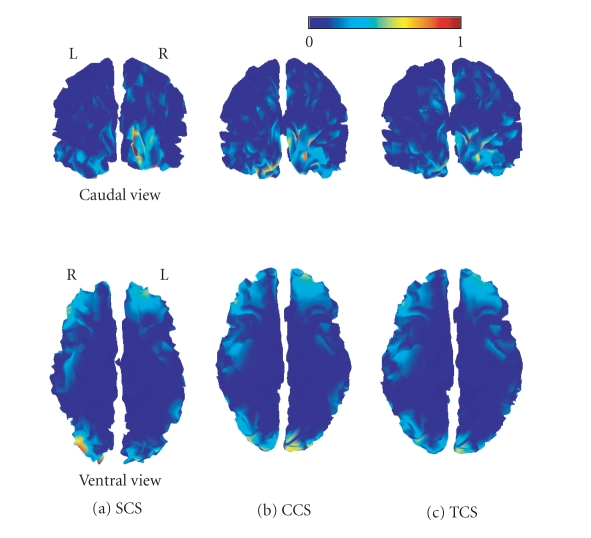
Caudal (upper row) and ventral (lower row) views of the cortical source energy estimated at the peak of the M170 for each of the three anatomical models: SCS (a), CCS (b), and TCS (c). Maps have been normalized to their maximum.

The two types of event-related fields (faces and scrambled) were subtracted to isolate a face-specific effect occurring around 170 milliseconds after stimulation (“M170”). Figure 5 shows the MEG setup and the M170 component elicited. Average responses, over a time window from 150 to 190 milliseconds, were estimated using the three models described above. The resulting log evidences are shown in Table 1. Figure 6 shows the corresponding maps of peak responses (conditional expectations of source activity at the time bin containing the maximum response).

Although slightly different, the estimated responses all show very similar activation patterns, namely in inferior occipital gyri (mostly right) and bilateral orbitofrontal poles. It should be noted that our simple minimum-norm solution has favored superficial activity (a well-known property of minimum norm solutions); analyses of the same data using more realistic models (with multiple sensor and source covariance components) place the maximum response more ventrally in both the fusiform and orbitofrontal regions [17]. However, we used the simplest model because this is the most established and our focus here is on *differences* in the reconstructed activity.

The log evidences for the three models are relatively close. One can assess the differences (log ratios or Bayes factors) using the semantics proposed by Kaas and Raftery by analogy with classical inference [15, 18]. In this context, a Bayes factor of twenty means that the data are twenty times more likely to have been generated by one model relative to another (cf., of *P* -value of .05). A Bayes factor of twenty corresponds to a difference in log evidence of about three, which is the typical threshold one would use to declare that one model was better than another. Given that the differences among the log evidences for our models were about twelve, there is strong evidence that SCS is better than CCS and that CCS is better than TCS. However, one cannot generalize from a single illustrative example. In Section 3.4 , we present an extensive simulation study to assess quantitatively and statistically the difference between the three models.

### 3.4. Synthetic data

**Figure 7:**
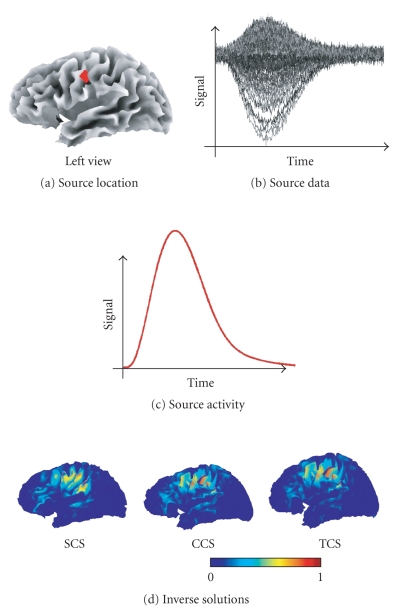
Example of a synthetic MEG dataset and its associated inverse solutions. Each map has been normalized to its maximum.

MEG data were simulated using the fine SCS mesh and the MEG setup described in Section 3.2 (see Figures 4(a) and 5, resp.). A hundred independent simulations were preformed, each using a single-extended source. For each simulation, the active source comprised a cluster of dipoles. Each cluster was constructed by selecting a random dipole and its nearest mesh neighbors, up to second order (including the nearest neighbors of the nearest neighbors). The cluster size was 7 ± 3 dipoles. Since the dipoles are spread uniformly over the cortical surface, this random dipole selection ensures that all brain regions were represented equally, over simulations. The activity of each source was modelled (over 321 time bins) with two gamma functions, whose parameters were selected randomly, subject to the constraint that the simulated activity reached a peak within time window modelled. Finally, after projection to sensor space, white Gaussian noise was added (SNR = 8 dB) (see Figure 7 for an example of simulated data).

The three models were inverted for each of the hundred simulated datasets. Since we know the true cortical activity, we supplemented our model comparison using the log evidence with the localization error (LE). LE is the distance between the true source and the dipole exhibiting the maximum estimated energy. This comparative metric complements the log evidence and speaks to the performance of the inversion in terms of the deployment of reconstructed activity, which is an important consideration in multisubject studies. To calculate LE for the SCS-(resp., CCS and TCS) based solution, we used the dipole on the coarse SCS (resp., CCS and TCS) which was closest to the truly activated source on the fine SCS.

### 3.5. Simulation results

**Figure 8:**
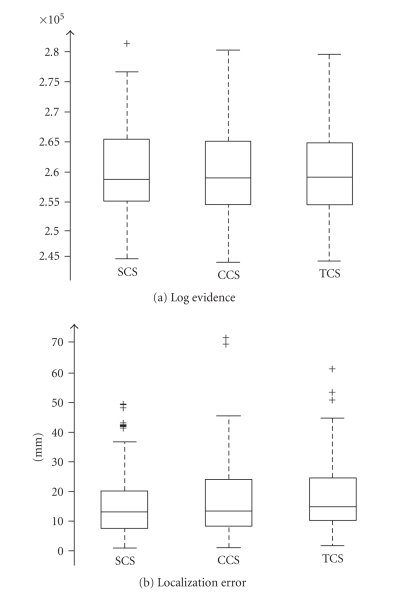
Whisker plots of the log evidences and LE values obtained with synthetic MEG data (similar to the example shown in Figure 7) for each of the three anatomical models (SCS, CCS, and TCS).

Figure 7 shows an example of synthetic data and the three solutions obtained for each mesh. Figure 8 shows the distributions (whisker plots) of the log evidence and LE over all simulations, for each of the three cortical models. The variance of the log evidences over source configurations is large. It is worth emphasizing here that a given log evidence has no meaning in itself. It only becomes meaningful when compared to the log evidence of another model applied to the same data.

The means of the log evidences, over models, show the same tendency as in the real-data example. Furthermore, the one-way within-dataset ANOVA on the log evidences was significant (*F* = 7.81; *P* < .0005***). Specifically, multiple comparisons with Bonferroni correction show that the only significant differences are between TCS and the two other models; suggesting that there is no demonstrable difference in the performance of the Bayesian inversion of the SCS and CCS models. Similarly, the one-way within-dataset ANOVA on the localization errors proved significant (*F* = 15.25; *P* < .0005***). Again, multiple comparisons with Bonferroni correction show that the only significant pairwise differences are between TCS and the two other models.

To summarize, the localizations based on the reference mesh (SCS) are significantly better than the ones based on the anatomically uninformed template mesh (TCS). Critically, when we transform the template mesh into the subjects anatomical space (CCS) there is no significant difference in localization error. Note that these results are obtained despite the fact that the SCS model should have been the best; since the synthetic data were generated using the similar, but with higher resolution, SCS model.

## 4. CONCLUSION

In this paper we have described a simple solution to the problem of reconstructing electromagnetic sources in a canonical anatomical space. Its simplicity rests on embedding subject specific anatomy into an extended forward model in a way that circumvents the need for cortical surface extraction. The forward model starts with a canonical cortical mesh, defined in a standard stereotactic space. The mesh is then warped into the subject's anatomical space. A conventional electromagnetic forward model is computed using the resulting warped mesh. The ensuing forward model is inverted using an established Bayesian scheme. Critically, the canonical mesh is warped using the inverse of the transformation used in conventional spatial normalization. This means that subject specific anatomy, encoded by the spatial transformation, can be derived from the subject's structural image using fully automated spatial normalization procedures that do not rely on high resolution or contrast.

The contribution of this work is twofold: first, conceptually we have formulated the problem of inter-subject anatomical variability as an explicit part of the forward model. This entails the notion of a canonical subject, whose cortical mesh is transformed anatomically to produce subject specific mesh. This places important constraints on individual meshes that enter the forward model; critically there must exist a diffeomorphic anatomical mapping between any subject and the canonical subject. We can exploit this constraint by always starting with the canonical mesh and warping it to match each subject. This has several fundamental advantages. First, it eschews the problems of cortical surface extraction from an individual's MRI; second it uses all the anatomical information in the MRI to construct a subject specific forward model (this information is not just confined to the cortical surface but includes all the information used in spatial normalization). Third, it ensures the cortical mesh is topologically valid (because it is derived under the diffeomorphism constraint). Finally, it enforces a standard solution space that facilitates inter-subject averaging and reporting. These standard spaces have proved very useful in fMRI.

The second contribution is the use of Bayesian model comparison, based on the model evidence or marginal likelihood to compare competing forward models. This enabled us to show that the models based on canonical meshes were at least as good as those based on individual cortical surface extraction. This provided a quantitative and principled way to explore model space and assess advances in model specification, of the sort addressed here.

We used Bayesian model comparison and localization error to evaluate the advantage of anatomically informed models (CCS) and to establish their construct validity in relation to conventional forward models based on cortical surface extraction (SCS). Importantly, our results do not show any systematic difference between the SCS and CCS models. This supports the idea that CCS is a sufficiently anatomically informed model to furnish a reasonable solution to the inverse problem. In other words, MEG data do not contain enough information about the fine-scale spatial configuration of sources to distinguish between the two models. Furthermore, TCS was significantly different from the other two models. This suggests that SCS or CCS should be used when possible. However, in the absence of structural MRI for any given subject, TCS remains a reasonable approximation, provided that it can be appropriately coregistered with the MEG data. The latter issue is crucial and will be addressed in a subsequent paper on optimizing use of template meshes, using only spatial information about sensor space (i.e., fiducials and head-shape data). Note finally, that we have focused on MEG and the use of a spherical head model. Although this approach could generalize to EEG in a straightforward way, we have not evaluated it yet in that context. This will require a careful analysis, due to the sensitivity of EEG to the geometry (and conductivity) of head tissues. This geometry is also subject specific and has been ignored here, because it is less important for MEG. However, a more realistic subject specific head model could be derived using the same approach used for the cortical mesh. This would entail using more realistic spheres or a boundary element model based on a canonical subject and warping it as described above. Again, Bayesian model comparison would enable us to assess the quantitative effect of realistic head tissue modeling.

To conclude, we have focussed on demonstrating the validity of the CCS model. This anatomically informed model has the twofold advantage of eschewing the need for cortical extraction and affording a one-to-one mapping with the canonical cortical surface. The latter is important for pooling results over subjects and reporting single subject or group localizations in the same stereotactic space. It also enables us to consider a full hierarchical model for multisubject analysis: namely, a unified inference scheme for group averages, instead of the conventional two-stage procedures (e.g., [17]). This includes, for example, incorporation of spatial priors on the MEG/EEG inverse solution based on normalized fMRI results from a group of subjects. This will be the focus of future work.

**Software note**

The algorithms described in this paper are available within SPM5 and can be downloaded from http://www.fil.ion.ucl.ac.uk/spm. It is worth emphasizing that the canonical cortical surface, given any subject's MRI, can be obtained automatically and robustly using the well-established spatial normalization schemes described in Section 2.2 SPM5 uses a unified forward model for anatomical deformations that includes tissue classification and inhomogeneity correction.

## Figures and Tables

**Table 1:** Log evidences obtained using the real MEG dataset for the three anatomical models.

	SCS	CCS	TCS
Log evidence	14084	14072	14058

## Footnotes

http://www.brainvisa.info[12]

By maximizing anatomical differences, we refer to cortical size and shape. The gender difference ensures a global difference in size. Moreover, differences in the shape and location of sulci are clearly visible (see Figure 4).
